# COVID-19 Vaccine Acceptance among Vulnerable Groups: Syrian Refugees in Jordan

**DOI:** 10.3390/vaccines10101634

**Published:** 2022-09-28

**Authors:** Qusai M. Talafha, Amal Al-Haidose, Ala Y. AlSamman, Saja A. Abdallah, Rasha Istaiteyeh, Wisam Nabeel Ibrahim, Ma’mon M. Hatmal, Atiyeh M. Abdallah

**Affiliations:** 1 Department of Economics, Faculty of Economics and Administrative Sciences, The Hashemite University, Zarqa 13133, Jordan; 2 Department of Biomedical Sciences, College of Health Sciences, QU Health, Qatar University, Doha 2713, Qatar; 3 University of Birmingham Medical School, Edgbaston Campus, Birmingham B15 2TT, UK; 4 Department of Medical Laboratory Sciences, Faculty of Applied Medical Sciences, The Hashemite University, Zarqa 13133, Jordan

**Keywords:** refugees, COVID-19, vaccine acceptance and hesitancy, vulnerable groups, Syrian, Jordan

## Abstract

Despite the wide distribution of COVID-19 vaccines, refugees remain last in line for the intake of vaccines. Syrian refugees in Jordan reach up to 700,000 registered and almost up to 700,000 unregistered refugees. This study aims to assess the willingness of Syrian refugees in Jordan to take the COVID-19 vaccine. Participants in the Zaatari refugee camp in Jordan were invited through social media to complete the survey between January and March 2022. A total of 230 refugees participated in our study, with almost half the participants of male gender. The majority of the participants had secondary school as their highest education level and were unemployed, being below the social poverty line. Interestingly, Syrian refugees showed a high vaccine acceptance rate, as 89.6% were willing to take the vaccine. Moreover, they showed high knowledge regarding the vaccine, the disease, and the virus. Our findings highlight the importance of knowledge and awareness of the COVID-19 vaccine to increase the acceptance rate. This is very important as refugees represent a vulnerable group to infection and complications and require close attention, especially with their significant numbers in Jordon and challenges of providing adequate vaccine supplies at their camps. We hope that, with proper dissemination of knowledge and awareness and with easy accessibility to the vaccines, it will ensure high immunization to reach herd immunity in Jordan.

## 1. Introduction

Since the outbreak of the pandemic in December 2019, COVID-19 vaccines have been extremely effective at preventing severe disease and reducing the risk of transmission [1]. However, continuing spread and emerging new variants of SARS-CoV-2 highlighted the need for urgent and equitable rollout of the vaccine to reach herd immunity [2,3]. In an attempt to overcome COVID-19 vaccine nationalism, the Vaccine Alliance (Gavi), the Coalition for Epidemic Preparedness Innovations (CEPI), and the WHO established the COVID-19 Vaccine Global Access (COVAX) facility to create a mechanism that will supply the vaccine globally, especially to low- and middle-income countries [4]. Unfortunately, this vision was not as successful as intended and vaccine rollout was much faster in high-income countries [5]. For example, in the early stages of vaccine distribution, USA secured 800 million, enough to vaccinate 140% of its population, by entering into multiple bilateral deals with six companies [6]. Similarly, the United Kingdom secured 270 million doses, which is enough to vaccinate 225% of its population [6]. The healthcare systems in low- and middle-income countries are overburdened and suffer from insufficient financial support [7]. Moreover, low- and middle-income countries host over 83% of worldwide refugees [8,9,10].

There are many displaced refugees seeking safety in Jordan from many conflicts happening in the Middle East. Because of two factors (Jordan’s stability and its geographical location), Jordan has become an important route to safety for many refugees from nearby countries such as Palestine, Iraq, and Syria. It is estimated that, in Jordan, there are 2 million Palestinian refugees, 67,000 Iraqi refugees, 15,000 Yemeni refugees, and 6000 Sudanese refugees [11]. These numbers show the Jordanian Government’s initiative to help refugees and it is no different when it comes to Syrian refugees. There are 658,000 Syrian refugees in Jordan; however, the United Nations High Commissioner for Refugees (UNHCR) estimates that the actual number could be up to 1.4 million [12]. Refugees require lots of support, which has placed a lot of pressure on the Jordanian Government in terms of living space and national resources. These include health care, education, funding, infrastructure, and resettlement resources. Refugee camps present unsanitary and cramped living conditions where diseases spread easily [13]. Despite many difficulties, especially when it comes to socio-political, environmental, and economic factors, Jordan has continued its support through different kinds of programmes. For example, as a response to the Syrian Crisis, two programmes were developed: the Sixth Regional Response Plan (RRP6) of UNCHR and the National Resilience Plan (NRP) of the government of Jordan [12]. This support was continued in the government’s response to the pandemic.

On 14 January 2021, Iraqi and Syrian refugees in camps began receiving their first doses of the COVID-19 vaccine under government guidelines [11]. Therefore, there is a certain level of accessibility when it comes to the COVID-19 vaccine for Syrian refugees. In addition, since the beginning of the pandemic, the government has indicated its intention to not discriminate against refugees irrespective of nationality or residency status when it came to vaccine prioritisation criteria [8]. As a result, the prioritisation criteria for vaccination has been the same for Syrian refugees as it has been for the rest of the Jordanian population, starting with healthcare workers, older people, and then people with underlying health conditions [14]. Understanding these facts presents us with the dilemma that some Syrian refugees may not be vaccinated because of their own hesitancy and we want to research the reasons for this, rather than vaccine accessibility. Perhaps by understanding the cause of their hesitancy, more initiatives can be put in place that target their concerns and lead to a higher acceptance of the COVID-19 vaccine. Even though, within UNHCR camps, positive COVID-19 cases are at around 1.6%, as compared with the general Jordan population, which is at 3% [8], the need for Syrian refugees to be vaccinated for their own safety and public health safety must not be undermined. It is important to highlight that the real infection rate in these refugee camps could be higher as case reporting is limited owing to a 14-day work ban for those who are infected [15].

## 2. Materials and Methods

### 2.1. Study Design and Participants

The study design is a descriptive cross-sectional web-based study. Participants over 18 years old in the Zaatari refugee camp (*n* = 400) were invited through social media randomly to complete the survey during January to March 2022, with 231 participants completing the survey (response rate 58%). Data were collected anonymously and confidentiality of information was guaranteed. Ethical approval was granted by the Ministry of Interior-Jordan. Informed consent was obtained from all participants after they were given detailed information about the study. Refugees had the right to refuse or withdraw from the study at any time without affecting their access to healthcare or social benefits.

### 2.2. Instrument

The instrument used in this study was originally developed and validated in a study conducted in USA, then the instrument was slightly modified and translated to Arabic and used in Qatar [16,17]. Syrian refugees are all fluent in Arabic as it is their first language. Therefore, in our study, we used same tool used in the study from Qatar [17]. To assess the Arabic questionnaire’s clarity among our participants, a pilot study was conducted among 10 refugees from the same camp. These participants were not included in the final analysis. The survey instrument consisted of eight sections that include 48 questions. The first part of the survey covers demographic questions. Intention to accept the COVID-19 vaccine was assessed using the following statement “Once COVID-19 vaccine is made publicly available”, and the participant can choose either “I would vaccinate myself” or “I would not vaccinate myself”. Then, we assessed the knowledge about COVID-19, preventive methods, transmission, and treatment among our participants by asking true and false questions, and the sum of correct answers was used as a knowledge score. The score ranged from 0 to 7 points, as for each right answer, a point was granted. The survey measures multiple factors towards COVID-19 vaccine attitudes that we hypothesized could influence vaccine acceptance, including the length of vaccine developing; efficacy of the vaccine (range includes 50%, 75%, and 99% vaccine effectiveness); vaccine side effects; and previous exposure to COVID-19 infection of either one’s self, family member, or friend. Moreover, we hypothesized that participants’ attitudes toward other vaccines, especially the flu vaccine, would be an important factor in acceptance of the COVID-19 vaccine.

### 2.3. Statistical Analysis

The results were analysed using the Statistical Package for the Social Sciences (SPSS) program, version 26 (IBM Corporation, New York, NY, United States). We measured the characteristics of survey participants using descriptive analysis and cross tabulation. Chi-squared tests were used to evaluate all categorical variables including factors that might influence willingness to take the vaccine. *p*-values were calculated for each independent variable. The differences in the knowledge scores between those participants who are willing to take the vaccine and those who are not willing were determined by *t*-tests.

## 3. Results

### 3.1. Sociodemographic Characteristics

A total of 230 responses were collected from refugees’ camps in Jordan; the number of female respondents was 130 (56.5%), while 100 (43.5%) respondents were males. According to the age of respondents, the majority were from the age group 18–24 years old, forming 47.4% of the whole sample, while the elderly group aged more than 46 years formed 5.7% of the whole respondents. Moreover, (68.7%) of respondents do not work and (70.9%) of respondent had an income less than 150 JD (Table 1).

### 3.2. Vaccine Acceptance

The question on willingness to take the vaccine was answered by all of the respondents (*n* = 230). In this study, 89.6% of participants agreed to be vaccinated against COVID-19, while 10.4% said “I would not be vaccinated”. When we posed the same question about vaccinations for their children, 121 out of 230 participants indicated they had children, and 13.9% of them claimed they would not vaccinate their children. The biggest worries for the people who said they would not take the vaccine were safety and effectiveness (33.3% and 29.2%, respectively). Moreover, parents indicated that the safety and effectiveness was the main reason to not give the vaccine to their children (78.1% and 12.5%, respectively). There were no significant differences between males and females in terms of willingness to vaccinate against COVID-19. Similarly, age and income were not associated with vaccine acceptance (data not shown).

### 3.3. Level of Knowledge

Of the participants, 91% had high knowledge scores, while the remaining participants had low knowledge scores. There was a statistically significant relationship between knowledge level among participants and their attitude to having the vaccine (Table 2). Participants with a high level of knowledge about COVID-19 tended to vaccinate more than participants with a low level of knowledge.

### 3.4. Attitudes and Beliefs toward COVID-19 Disease and Vaccine

**Figure 1:**
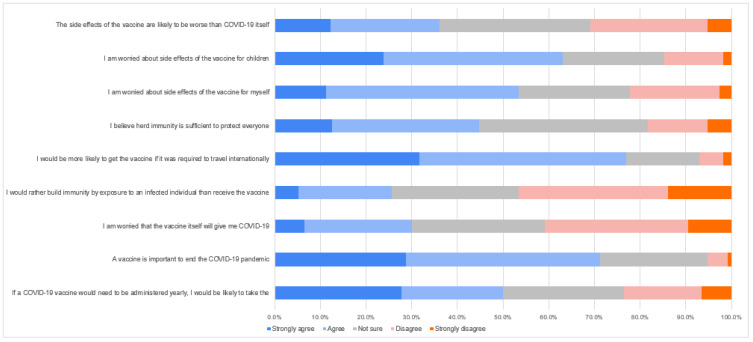
Attitudes and beliefs toward COVID-19 disease, vaccination, and immunity using a five-point Likert scale.

The attitudes and beliefs of the respondents regarding COVID-19 disease, vaccination, and immunity using a five-point Likert scale are shown in Figure 1. In this study, 83.9% agreed or strongly agreed with the statement “Is vaccination important as a protection from serious diseases?”. Moreover, 71.3% of participants said that they “agree and strongly agree” with the statement “vaccination against COVID-19 is important to end the pandemic”. Further, 63.0% of participants answered “agree” or “strongly agree” to the statement “I am worried about the side effects of the vaccine for my children”, and about 53.5% were worried about the side effect for themselves. According to participant beliefs, 44.8% of participants agreed that herd immunity is sufficient to protect everyone, while 18.2% of the participants did not agree with this notion and 37% of the participants were neutral. Moreover, 77% of the participants agreed with the vaccination requirement for travelling abroad, while 6.9% disagreed. A further 40% of the respondents demonstrated that their comfortability does not necessarily depend on the vaccines developed in America or Europe compared with other countries or regions, while 34% of the respondents were neutral and 27% confirmed they are more comfortable if the vaccine is developed in these countries. Finally, 46.5% of the respondents did not think they would develop more immunity if they became infected from another person compared with taking the vaccine.

### 3.5. Determinants Influencing COVID-19 Vaccine Acceptance

**Figure 2:**
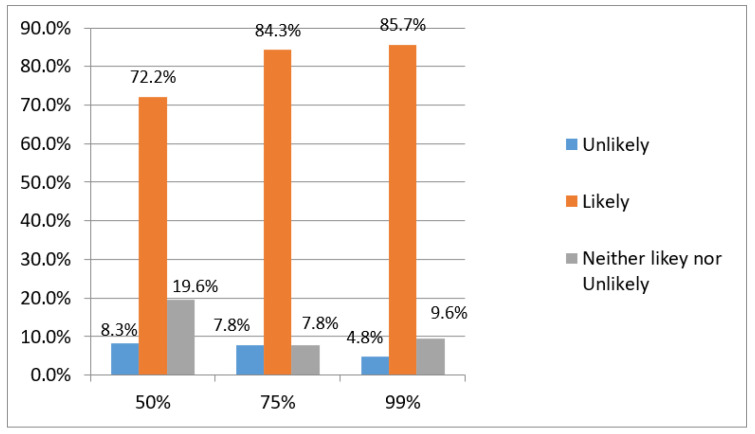
Different levels of vaccine efficacy and participants’ responses on how likely they were to be vaccinated.

Table 3 depicts the association between influencing factors impacting vaccine acceptance. Respondents who thought it was important to get an annual influenza vaccination were less likely to reject the COVID-19 vaccine (*p* < 0.001). However, 6% of those who indicated it was important to obtain the influenza vaccine said they would not get the COVID-19 vaccine. In terms of the information and news on COVID-19, although 112 out of 230 (48.7%) participants indicated that they depended on professional sources (local health authority, primary doctor, and WHO) when deciding whether to take the vaccine, there were no significant differences between the source of information and willingness or unwillingness to be vaccinated. Previous COVID-19 exposure did not not affect the respondents’ willingness to take the vaccine. The majority of participants were concerned about the rushed speed of COVID-19 vaccine development. Finally, the majority of the respondents stated they were more inclined to be vaccinated when the percentage efficacy increased (Figure 2).

## 4. Discussion

In this study, we aimed to identify factors that contribute to the willingness to take the COVID-19 vaccine among Syrian refugees in Jordan. In our survey, 230 refugees participated, with approximately equal percentages of both genders. The majority of participants were from the age group of 18–24 years old. The majority of participants were singles, with the highest education level being secondary school. In addition, the majority of refugee participants were either unemployed or their income per month was less than 150 JD (almost 200 USD). This is under the social poverty line index for Jordan, which is 323 JD [18]. While financial difficulty may not seem to be directly related to not accepting the vaccine, as it is given free of charge, it has an impact on many other aspects of their lives. For example, their living conditions may result in an inaccessibility to reliable sources of information, which, as we discussed before, leads to misinformation and fear regarding the vaccine. Interestingly, Syrian refugees showed a high vaccine acceptance rate, as 89.6% were willing to take the vaccine. Overall, respondents of this study had a positive acceptance rate towards a vaccine against COVID-19. Syrian refugees in Lebanon showed a similarly high acceptance rate as well [19]. This acceptance rate is also higher than that of the local Jordanian population, as a study conducted in Jordan found that 36.3% refused to take the vaccine [20]. Quite a large percentage of the local population were hesitant to take the vaccine. Alternatively, Syrian refugees show high willingness to vaccinate their children, with only 13.9% unwilling to do so, which is very low compared with other studies reporting high unwillingness of parents from Arab countries to vaccinate their children [21,22,23,24,25,26].

We assessed refugees’ knowledge regarding the COVID-19 vaccine, virus, and disease by asking seven true or false questions and calculating correct responses. Among our refugee participants, knowledge was an important factor associated with vaccine acceptance. According to our findings, 91% of our participants had a “high knowledge” score about COVID-19 disease and vaccine. Understanding disease mechanism and possible side effects of the vaccine was shown to be important in other groups in Jordan and other Middle East countries. A study performed in three Middle Eastern countries (Jordan, Saudi Arabia, and Kuwait) found that participants had moderate overall COVID-19 knowledge, and had better knowledge about disease prevention and control [27,28]. Higher education participants had a good level of understanding, which could be linked to their level of education. However, this is not representative of the whole population. It is expected that knowledge among those with a lower education level or in situations that does not allow them to get an adequate education may be considerably low. This notion could be of particular relevance in vulnerable groups including refugees and those under the poverty line. This is because of their access to a reliable source of information. For example, a correlation between COVID-19 vaccine hesitancy and reliance of social media as the main source of information about COVID-19 has been found [12]. Through unreliable sources, misinformation about COVID-19 and its vaccine has increased. Another possible reason for vaccine hesitancy is the conspiracy theories associated with it. These include the beliefs that the vaccine is used to inject citizens with microchips and that it is meant for family control by causing infertility, which was believed by 27.7% of the Jordanian population in a recent study [20]. These theories spread fear and paranoia, which can prevent people from getting the vaccine, even if they concerned with the risk of getting the disease. It can also lead to parents refusing to give their child this vaccine for the same belief.

One of the important factors for a high vaccine acceptance rate in Jordan is that the UNHCR in Jordan has been working closely with the Jordanian Ministry of Health to roll out the vaccination campaign. Many mobile vaccination units were opened in different locations in Jordan. This partnership is focused on raising the awareness of refugees about the benefits of taking the vaccine and encouraging them to register. “The success of the vaccination campaign is very much connected to the Government’s decision to include all persons on Jordanian territory, nationals, and refugees”, said a UNHCR Jordan Representative [29]. In February 2022, UNHCR-Jordan has announced that vaccination efforts are to continue, with over 90% of Syrian refugees in camps and 50% in urban areas having been vaccinated [30]. Moreover, in early March 2022, over 48,000 camp-based refugees and 13,000 urban refugees had been vaccinated in UNHCR Registration Centres (RCs) and community centres. The results show that the COVID-19 vaccination rate among Syrian refugees is over 90% in camps and 50% in urban areas (18 years and above) [9]. Moreover, as part of the UNHCR’s ongoing outreach efforts on the COVID-19 vaccination program, four focus group discussions (FGDs) with over 60 refugees of different nationalities were held in Amman (Nuzha), Zarqa, Irbid, and Mafraq, adhering to health and safety measures. Participants received information on the COVID-19 vaccination program and registration process on the government platform, as well as information on specific questions such as side effects and chronic diseases [30].

Our study has a few limitations. First, we had difficulty in reaching out to the households at Zaatari camp, as people were afraid that the data collected might inform local public health authorities in Jordan on their willingness/unwillingness to take the vaccine and its ramifications on their survival livelihoods. Another limitation was the difficulty in accessing the camps, owing to security and privacy matters for the refugees themselves. In addition, the current study includes a small sample size, so the finding in this study cannot be generalized to all refugees in Jordan or other countries in the Middle East. Moreover, there is a tendency for bias as the participants who were willing to participate in the study were more likely to be literate to fill in the online instrument, while illiterate refugees and others with disabilities or diseases would unlikely be available to participate in this study. In addition, refugees might show bias in answering the instrument question to fulfill their commitment toward the official rules and regulations by the government.

## 5. Conclusions

In this representative refugee cohort, our findings highlight the importance of knowledge and awareness of the COVID-19 vaccine to increase the acceptance rate. This is very important as refugees constitute a significant proportion of the Jordan population. Moreover, Syrian refugees showed a higher acceptance compared with the local Jordanian population. However, they may face some challenges in getting the vaccine. Proper dissemination and easy accessibility will ensure high immunization to reach herd immunity in Jordan.

## Figures and Tables

**Table 1:** Sociodemographic of refugees’ respondents.

Demographic	Variable Category	*n*	%
Gender	Male	100	43.5
	Female	130	56.5
Age	18–24 years	109	47.4
	25–31 years	41	17.8
	32–38 years	38	16.5
	39–45 years	29	12.6
	46+ years	13	5.7
Education level	Less than secondary school	120	52.2
	Diploma	35	15.2
	Bachelor	55	23.9
	Master	4	1.7
	Doctorate	2	0.9
	Other	14	6.1
Marital status	Single	104	45.2
	Married	119	51.7
	Divorced	1	0.4
	widow	6	2.6
Work	Yes	72	31.3
	No	158	68.7
Income	Less than 150	163	70.9
	151–250	50	21.7
	251–350	14	6.1
	351–450	1	0.4
	650+	2	0.9

**Table 2:** Relationship between COVID-19 knowledge score and willingness to vaccinate.

Knowledge Score	Would Vaccinate *n* (%)	Would Not Vaccinate *n* (%)	X^2^	*p*-Value
High (1–2.6)	191 (90.9)	19 (9.1)	4.972	0.04
Low (2.61–3)	15 (75)	5 (25)		

**Table 3:** Relationship between factors and determinants influencing acceptance and willingness to vaccinate.

Question	Answers	Would Vaccinate *n* (%)	Would Not Vaccinate *n* (%)	Multinomial Logistic Regression Analysis		
				Odds ratio	95% CI	*p*
What is your primary source of information regarding COVID-19?	Professional sources	104 (93%)	8 (7%)	0.95	0.65–1.35	0.76
	Unprofessional sources	94 (89%)	12 (11%)	4.29	0.77	0.09
	Leaders	8 (67%)	4 (33%)	Reference		
How important is it for you to get the flu vaccine every year?	Important	142 (94%)	9 (6%)	5.94	1.65–21.3	0.006
	Somewhat important	38 (88%)	5 (12%)	2.47	0.64–950	0.18
	Not important	26 (72%)	10 (28%)	Reference		
To what extent do you follow the news of COVID-19?	Follow	81 (93%)	6 (7%)	1.38	0.38–5.05	0.61
	Neither follow nor do not follow	56 (90%)	6 (10%)	0.94	0.27–3.23	0.92
	Do not follow	69 (85%)	12 (15%)	Reference		
I worry that the rushed pace of testing for a COVID-19 vaccine will fail to detect potential side effects.	Agree	113 (89%)	14 (11%)	1.58	0.31–7.96	0.57
	Neither agree nor disagree	74 (91%)	7 (9%)	1.73	0.31–9.50	0.52
	Disagree	19 (86%)	3 (14%)	Reference		
If COVID-19 vaccine is available and said it would protect 50% of those who take it, how likely you would take the vaccine?	Likely	155 (93%)	11 (7%)	7.34	2.04–26.37	0.002
	Neither likely nor Unlikely	39 (87%)	6 (13%)	5.22	1.21–22.51	0.027
	Unlikely	12 (63%)	7 (37%)	Reference		
The side effects of the vaccine are likely to be worse than COVID-19 itself.	Likely	70 (84%)	13 (16%)	0.32	0.08–1.17	0.32
	Neither likely nor Unlikely	70 (92)	6 (8%)	1.10	0.27–4.45	1.10
	Unlikely	66 (93)	5 (7%)	Reference		

## Data Availability

Not applicable.
